# Toxinology and Pharmacology of Snake Venoms

**DOI:** 10.3390/toxins15030212

**Published:** 2023-03-10

**Authors:** Igor Križaj

**Affiliations:** Department of Molecular and Biomedical Sciences, Jožef Stefan Institute, SI-1000 Ljubljana, Slovenia; igor.krizaj@ijs.si

Evolution endowed snakes with the ultimate weapon: venom [1]. With it, several hundred species of venomous snakes can kill or weaken their victims to prevent them from escaping. Snakes get closer to humans and cause more harm and more deaths than any other venomous animal, including spiders and scorpions [2]. Snake venom can be particularly dangerous for the circulatory, nervous or muscular systems of humans [3]. The increased sensitivity of analytical instruments and the development of new techniques over the last two decades, such as transcriptomics and proteomics, have allowed us to analyze the structures and functions of venom components of rare snake species and to identify novel minor snake venom constituents [4,5]. As a result, the number of polypeptides identified in snake venoms is increasing dramatically. The unraveled biochemical composition, genomics and proteomics of toxins and venoms have deepened our understanding of their interaction with organisms, most importantly with humans. Their modes of action are better understood, which is opening the door for their application as molecular tools, diagnostic or therapeutic agents, including the development of antidotes [6]. Snake venom research influences various areas of life and biomedical sciences. It is tightly intertwined with biochemistry, molecular biology, genetics, pathophysiology, pharmacology and a rapidly developing field of clinical toxinology. The latter deals with understanding and managing the medical effects of toxins on the human body. Given the huge impact of deaths and disabilities due to snake venom poisoning around the world and the potential of venoms in the development of drugs against various diseases, soaring of this field of research is certain. This Special Issue of *Toxins* brings a selected set of articles addressing biochemical, therapeutic and evolutionary aspects of snake venom research.

Laxme et al. present a comparative venomics study of two Daboia snakes, *D. palaestinae* and *D. russelii* (‘The Middle Eastern Cousin: Comparative Venomics of *Daboia palaestinae* and *Daboia russelii*’) to unravel the factors responsible for the much larger medical relevance of the latter snake. Their findings highlight the differences in the venom composition, function and toxicity of the two Daboia species, supporting the thesis that phylogenetic relatedness of snakes cannot readily predict venom protein composition or function. Op den Brouw et al. also studied Middle Eastern Snakes (‘Extensive Variation in the Activities of Pseudocerastes and Eristicophis Viper Venoms Suggests Divergent Envenoming Strategies Are Used for Prey Capture’). They report a large variation in composition and activity of the venoms of two desert vipers *Pseudocerastes urarachnoides* and *Eristicophis macmahoni*, likely the consequence of the prey specificity. An important message of these two papers is that the phylogenetic relatedness of snakes does not allow confident predictions about the venom protein composition or pharmacology of venom proteins, and thus the efficacy of paraspecific antivenom therapy. This is experimentally demonstrated by Kurtović et al. (‘Intravenous *Vipera berus* Venom-Specific Fab Fragments and Intramuscular *Vipera ammodytes* Venom-Specific F(ab’)2 Fragments in *Vipera ammodytes*-Envenomed Patients’), who compared, in their study, the clinical efficacy of the treatment of the *V. ammodytes*-envenomed patients with either a specific, *V. ammodytes* antivenom or a paraspecific, *Vipera berus* antivenom. Paraspecific serotherapy was not effective in suppressing thrombocytopenia, while the progression of rhabdomyolysis and neurotoxicity was not prevented at all. A similar conclusion could also be drawn from the study by Huynh et al. in this paper collection (‘The Effect of Australian and Asian Commercial Antivenoms in Reversing the Post-Synaptic Neurotoxicity of *O. hannah*, *N. naja* and *N. kaouthia* Venoms In Vitro’); nevertheless, if it is the only one available, the paraspecific antivenom could still be of great value.

Toxin-encoding genes are among the most dynamically evolving gene families in nature. Detailed studies of mechanisms of their molecular evolution can provide knowledge that is broadly applicable, for example, in answering questions about the origin of novel protein functions. In their study (‘The Target Selects the Toxin: Specific Amino Acids in Snake-Prey Nicotinic Acetylcholine Receptors That Are Selectively Bound by King Cobra Venoms’), Chandrasekara et al. illuminate the selection pressure exerted by a specialist prey organism on the evolution of lineage-selective toxins. Finally, yet importantly, the present Special Issue offers an original insight into the evolution of snake venom metalloproteinases (SVMPs) that urged a redefinition of the classification of these snake venom proteins. Based on the gene structure of a disintegrin-like/cysteine-rich protein from the venom of the nose-horned viper, in their paper (‘Genomic Confirmation of the P-IIIe Subclass of Snake Venom Metalloproteinases and Characterisation of Its First Member, a Disintegrin-Like/Cysteine-Rich Protein’) Požek et al. proposed the introduction of a new P-IIIe subclass of SVMPs. 

## Data Availability

Not applicable.
